# Supplementary material for: Spin Wave Electromagnetic Nano‐Antenna Enabled by Tripartite Phonon‐Magnon‐Photon Coupling
Source: Adv Sci (Weinh). 2022 Jan 19;9(8):2104644. doi: 10.1002/advs.202104644 (PMC8922090; doi:10.1002/advs.202104644)
Supplement: Supplementary file 1 — Supporting Information [file ADVS-9-2104644-s001.pdf]

ADVANCED SCIENCE
Open Access

Supporting Information

for *Adv. Sci.*, DOI 10.1002/advs.202104644

Spin Wave Electromagnetic Nano-Antenna Enabled by Tripartite Phonon-Magnon-Photon Coupling

*Raisa Fabiha, Jonathan Lundquist, Sudip Majumder, Erdem Topsakal, Anjan Barman and Supriyo Bandyopadhyay**

ADVANCED SCIENCE
Open Access

Supporting Information

for *Adv. Sci.*, DOI: 10.1002/advs.202104644

Spin Wave Nano-Antenna Enabled by Tripartite Phonon-Magnon-Photon Coupling

Raisa Fabiha[1], Jonathan Lundquist[1], Sudip Majumder[2], Erdem Topsakal[1], Anjan Barman[2] and Supriyo Bandyopadhyay[1]*

Supporting Information: Spin Wave Nano-Antenna Enabled by Tripartite Phonon-Magnon-Photon Coupling

Raisa Fabiha[1], Jonathan Lundquist[1], Sudip Majumder[2], Erdem Topsakal[1], Anjan Barman[2] and Supriyo Bandyopadhyay[1*]

[1]Department of Electrical and Computer Engineering, Virginia Commonwealth University, Richmond, VA 23284, USA

[2]Department of Condensed Matter Physics and Material Sciences, S. N. Bose National Center for Basic Sciences, Block JD, Sector III, Salt Lake, Kolkata 700 106, India

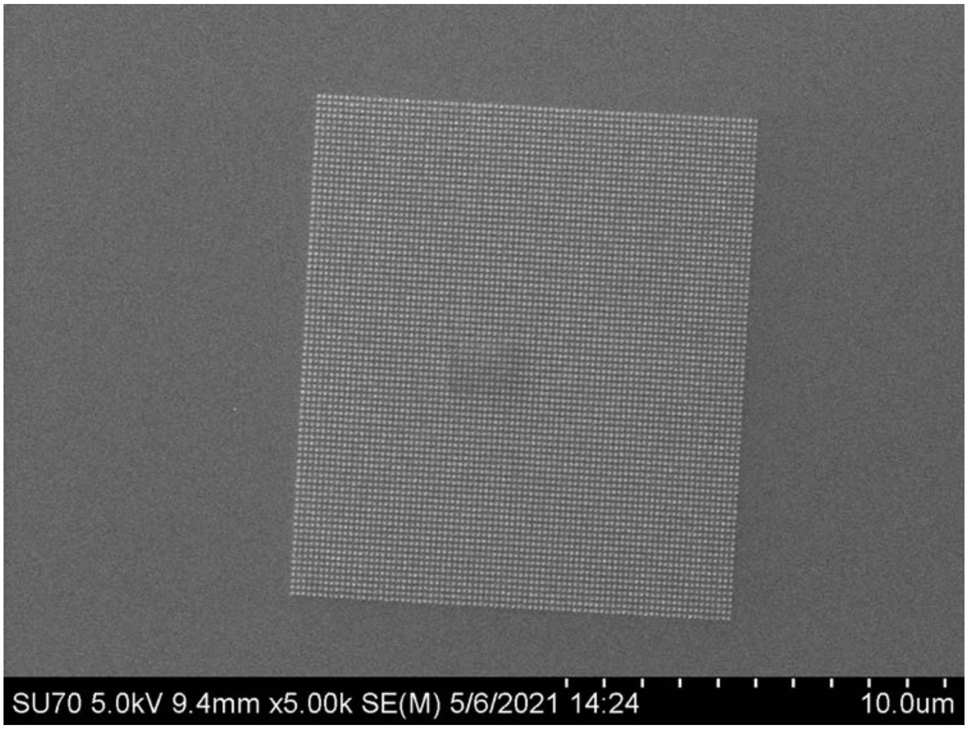

Fig. S1: Low magnification scanning electron micrograph of a square containing 80×80 nanomagnets. The antenna is made up of 200 such squares.

The rest of the Supplementary Material contains the data for the cases when the SAW exciting frequency is 14 GHz, 30 GHz and 35 GHz. The data were acquired only in orientation 2 when the microwave power

[*] Corresponding author. Email: sbandy@vcu.edu

source was connected between terminals 5 and 7. The propagation of the SAW in this case was along the major axis of the elliptical nanomagnets.

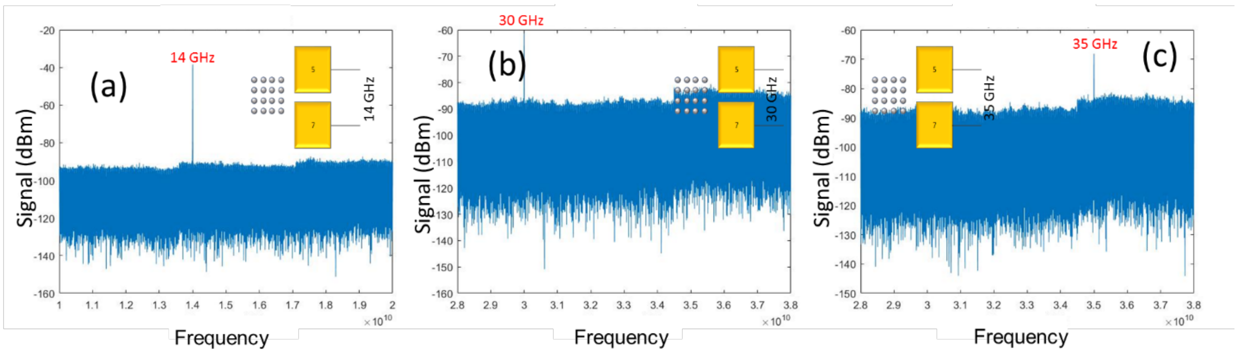

Fig. S2: Spectrum of received power at the horn antenna when the antenna was placed 36.8 cm from a sample. The sample was excited in orientation 2, i.e. the signal was fed to electrodes 5 and 7. (a) Excitation (SAW) frequency is 14 GHz, (b) Excitation (SAW) frequency is 30 GHz, and (c) Excitation (SAW) frequency is 35 GHz.

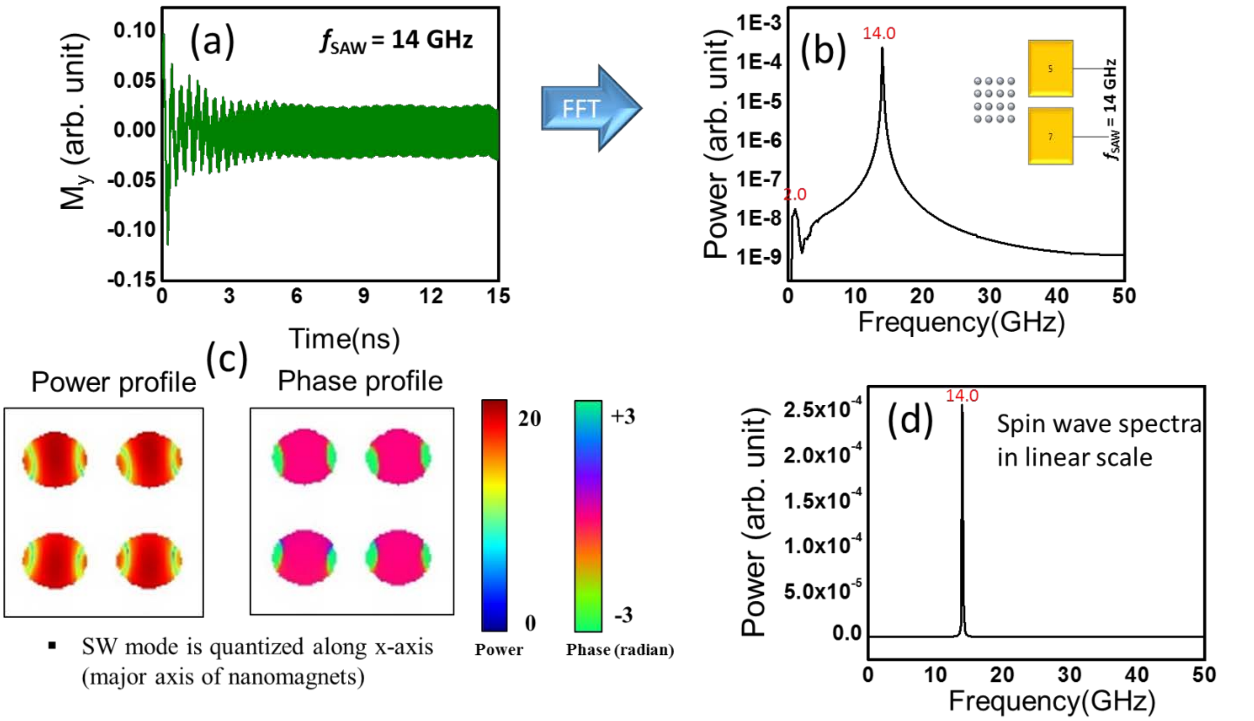

Fig. S3: (a) Calculated oscillations in the magnetization component parallel to the minor axis of the elliptical nanomagnets (hard axis) when the exciting SAW frequency is 14 GHz. The sample is excited in orientation 2 where the direction of propagation of the SAW is along the major axes of the elliptical nanomagnets. (b) The power spectrum of the oscillations in log linear scale where the dominant peak is at the excitation frequency. The next dominant peak has a power amplitude almost 4 orders of magnitude smaller. (c) The power and phase profiles of the spin waves in the 14 GHz (dominant frequency) mode showing that the wave is quantized along the major axis (also direction of propagation of the SAW). (d) The power spectrum of the oscillations in linear scale.

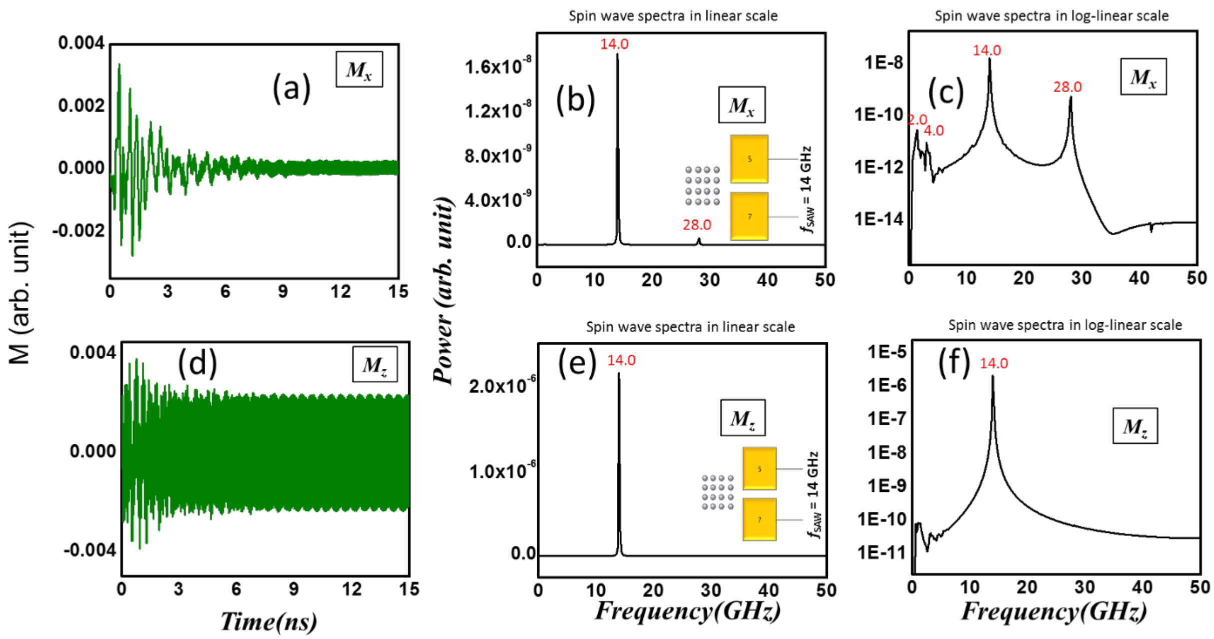

Fig. S4: (a) Calculated oscillations in the magnetization component parallel to the major axis of the elliptical nanomagnets (easy axis) when the exciting SAW frequency is 14 GHz. The sample is excited in orientation 2 where the direction of propagation of the SAW is along the major axes of the elliptical nanomagnets. (b) The power spectrum of the oscillations in linear scale where the dominant peak is at the excitation frequency, but there is a side band at twice the excitation frequency whose power is at least fifty times smaller. (c) The power spectrum in log linear scale showing some side bands. The power in the dominant oscillation mode of M_x (at 14 GHz) is more than 4 orders of magnitude smaller than the power in the dominant oscillation mode of M_y at 14 GHz shown in Fig. S2. (d) Oscillations in the normal-to-plane magnetization component. (e) The power spectrum of the oscillations in linear scale where the dominant peak is at the excitation frequency of 14 GHz. (f) The power spectrum in log linear scale showing some side bands. Again, the power in the dominant oscillation mode of of M_z (at 14 GHz) is more than 2 orders of magnitude smaller than the power in the dominant oscillation mode of M_y at 14 GHz shown in Fig. S2.

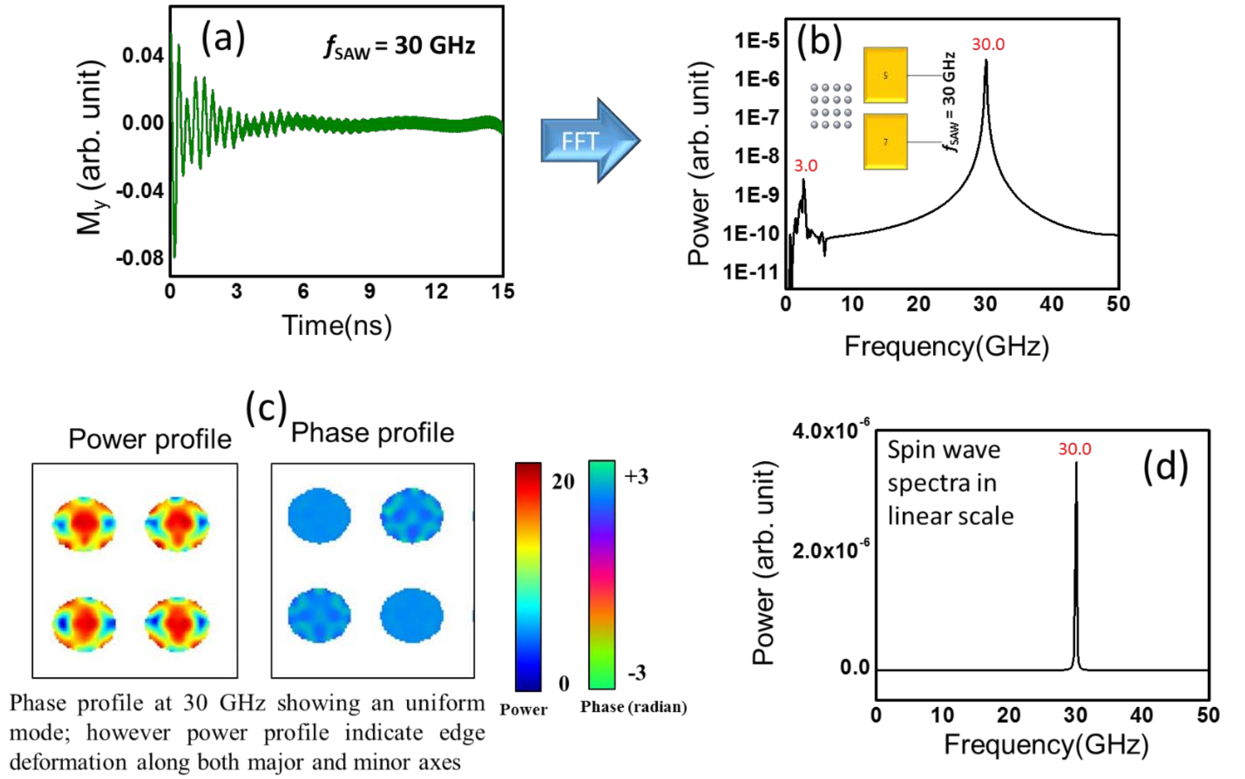

Fig. S5: (a) Calculated oscillations in the magnetization component parallel to the minor axis of the elliptical nanomagnets (hard axis) when the exciting SAW frequency is 30 GHz. The sample is excited in orientation 2 where the direction of propagation of the SAW is along the major axes of the elliptical nanomagnets. (b) The power spectrum of the oscillations in log linear scale where the dominant peak is at the excitation frequency. The next dominant peak at 3 GHz has a power amplitude almost 4 orders of magnitude smaller. (c) The power and phase profiles of the spin waves in the 30 GHz (dominant frequency) mode showing that the phase profile is uniform but the power profile has deformations along both major and minor axes of the elliptical nanomagnet. (d) The power spectrum of the oscillations in linear scale.

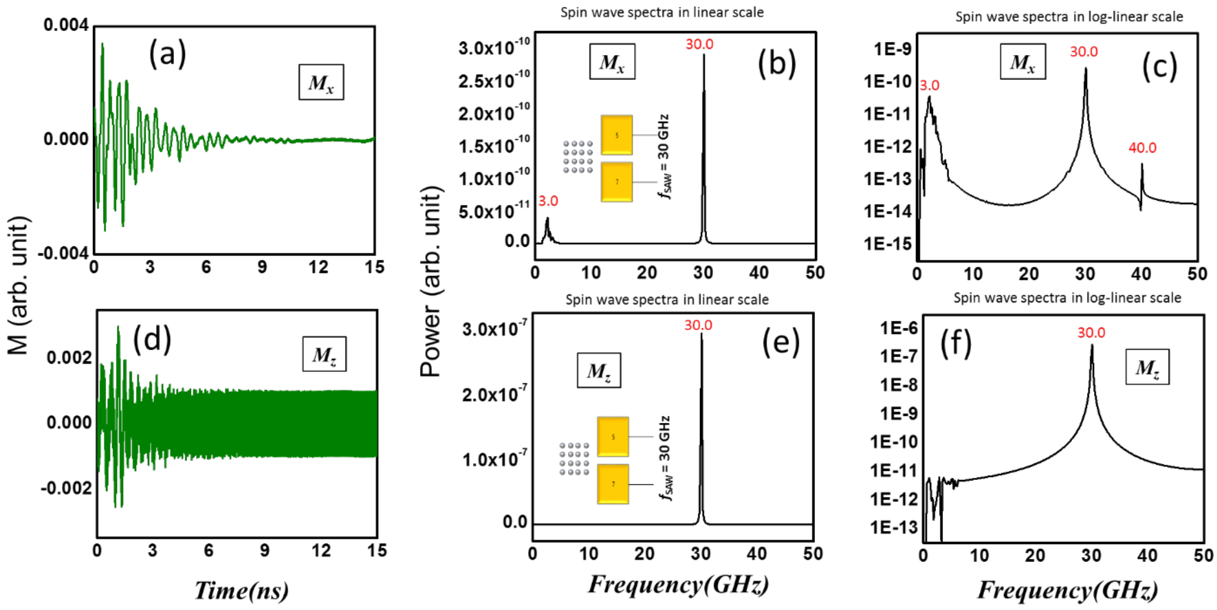

Fig. S6: (a) Calculated oscillations in the magnetization component parallel to the major axis of the elliptical nanomagnets (easy axis) when the exciting SAW frequency is 30 GHz. The sample is excited in orientation 2 where the direction of propagation of the SAW is along the major axes of the elliptical nanomagnets. (b) The power spectrum of the oscillations in linear scale where the dominant peak is at the excitation frequency, but there is a strong side bands at 3 GHz. The power in the side band is one order of magnitude smaller than that in the dominant mode, which occurs at the excitation frequency of 30 GHz. (c) The power spectrum in log linear scale showing another weaker side band at 40 GHz whose power is more than two orders of magnitude smaller than that of the dominant mode. The power in the dominant oscillation mode of M_x (at 30 GHz) is more than 4 orders of magnitude smaller than the power in the dominant oscillation mode of M_y at 30 GHz shown in Fig. S4. (d) Oscillations in the normal-to-plane magnetization component. (e) The power spectrum of the oscillations in linear scale where the dominant peak is at the excitation frequency of 30 GHz. (f) The power spectrum in log linear scale. The power in the dominant oscillation mode of of M_z (at 30 GHz) is about the same as the power in the dominant oscillation mode of M_y at 30 GHz shown in Fig. S4.

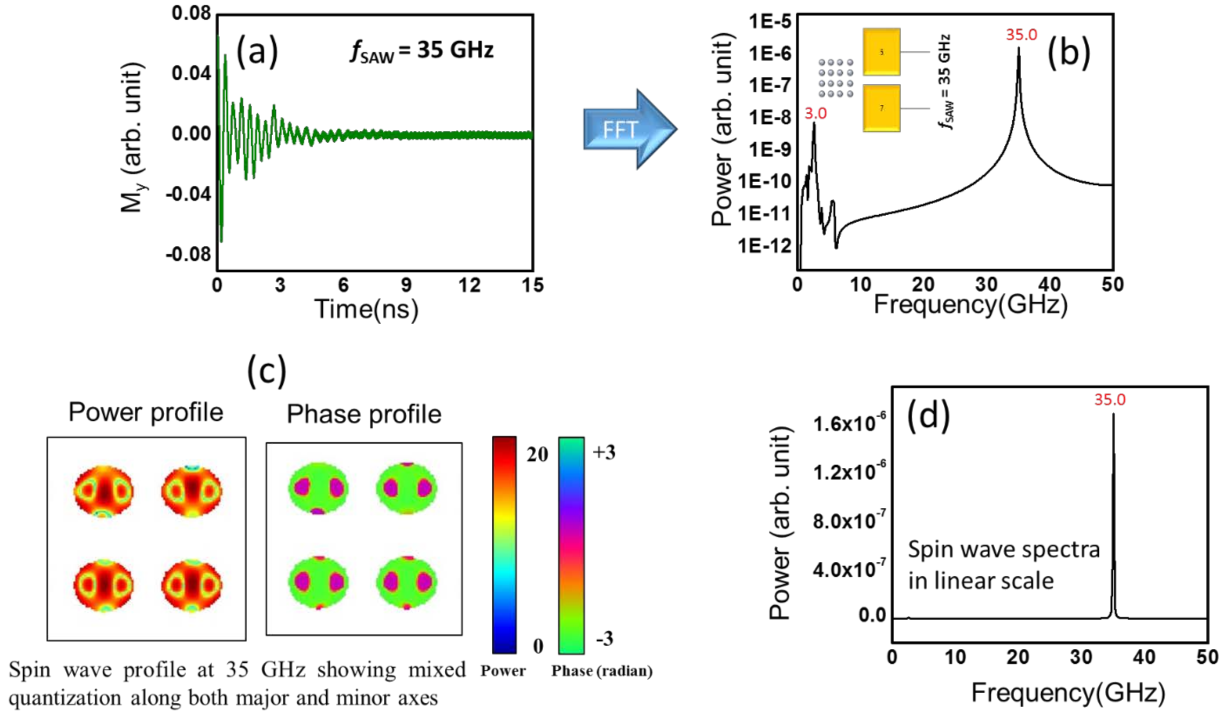

Fig. S7: (a) Calculated oscillations in the magnetization component parallel to the minor axis of the elliptical nanomagnets (hard axis) when the exciting SAW frequency is 35 GHz. The sample is excited in orientation 2 where the direction of propagation of the SAW is along the major axes of the elliptical nanomagnets. (b) The power spectrum of the oscillations in log linear scale where the dominant peak is at the excitation frequency of 35 GHz. The next dominant peak at 3 GHz has a power amplitude more than 2 orders of magnitude smaller. (c) The power and phase profiles of the spin waves in the 30 GHz (dominant frequency) mode showing mixed quantization along both major and minor axes of the elliptical nanomagnet. (d) The power spectrum of the oscillations in linear scale.

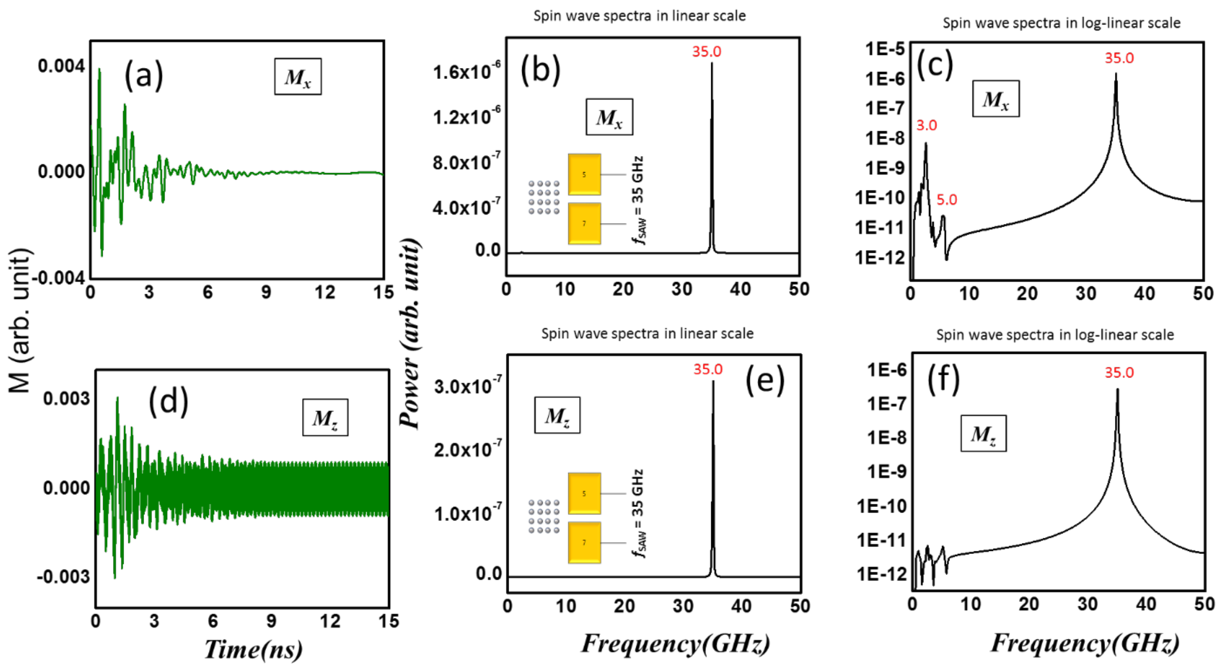

Fig. S8: (a) Calculated oscillations in the magnetization component parallel to the major axis of the elliptical nanomagnets (easy axis) when the exciting SAW frequency is 35 GHz. The sample is excited in orientation 2 where the direction of propagation of the SAW is along the major axes of the elliptical nanomagnets. (b) The power spectrum of the oscillations in linear scale where the dominant peak is at the excitation frequency. (c) The power spectrum in log linear scale showing a side band at 3 GHz whose power is more than two orders of magnitude smaller than that of the dominant mode. The power in the dominant oscillation mode of M_x (at 35 GHz) is about the same as the power in the dominant oscillation mode of M_y at 35 GHz shown in Fig. S6. (d) Oscillations in the normal-to-plane magnetization component. (e) The power spectrum of the oscillations in linear scale where the dominant peak is at the excitation frequency of 35 GHz. (f) The power spectrum in log linear scale. The power in the dominant oscillation mode of M_z (at 35 GHz) is slightly smaller than the power in the dominant oscillation mode of M_y or M_x at 35 GHz.

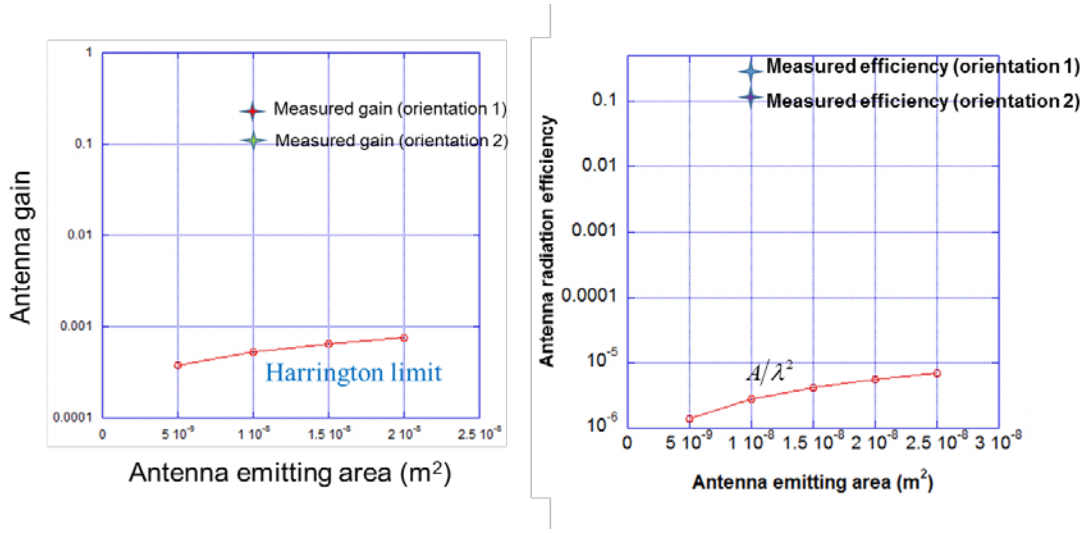

Fig. S9: Plots of antenna gain and radiation efficiency as a function of the antenna emitting area at frequency of 5 GHz (wavelength 6 cm), showing the theoretical limits and the measured quantities in both orientations.

On the validity of obtaining the power radiated by the nanomagnets by subtracting the received power from the control sample from the total received power

A sample has multiple constituents that can radiate electromagnetic waves – the nanomagnets, the contact pads, the wires, etc. That is why it is imperative to make a "control" sample which has all the peripherals, but not the nanomagnets. We have obtained the power radiated by the nanomagnets by subtracting the power radiated by the control sample from the actual received power, but this approach needs justification.

Let us call the intensity of the radiation received at any point in space from the nanomagnets I_1 and the intensity of radiation received from the peripherals I_2. Because the radiation is likely coherent, the total intensity at the receiving point is $I = I_1 + I_2 + 2\sqrt{I_1 I_2}\cos\theta$ where θ is the phase difference between the electric fields in the two beams at the receiving point. The last term is the interference term and implicit in the above expression is the assumption that both the sources and the detector are point sources and point detector respectively.

Even if we can view the sources as point sources, the receiving horn antenna is certainly not a "point". Its receiving area is much larger than the square of the wavelength. The value of θ is different at different regions of the horn's receiving area and when we spatially average over the receiving area, $\cos\theta$ will average out to its average value of zero approximately. In our case, the antenna's linear dimension is about 3 times larger than the wavelength at the lowest frequency of 5 GHz and hence the averaging may not be complete, but we believe that neglecting any interference effect is nonetheless a good approximation for us. That is why we are justified in neglecting the interference term and making the approximation that the total received power is the sum of the power due to the nanomagnets and everything else. This then allows us to do the background subtraction with the control sample.

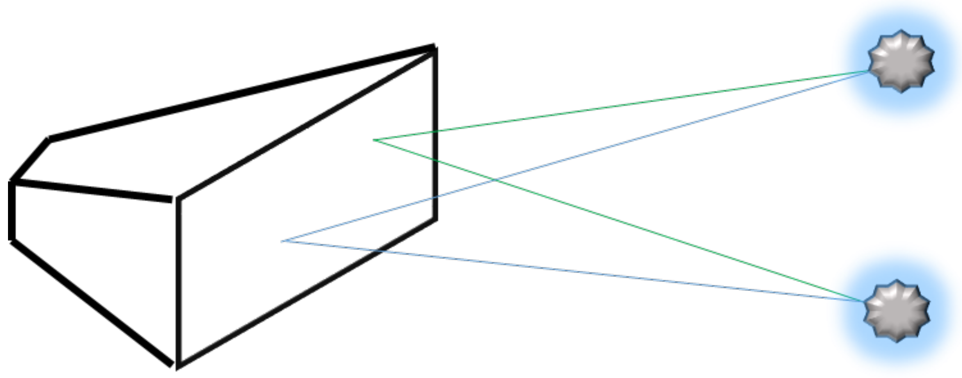

Fig. S10: Cancellation of interference of radiations from point sources received at an extended detector.
